# Complete Genome Sequences of Subcluster C1 Mycobacteriophages Blackbrain, Cactojaque, Kboogie, Trinitium, and YoungMoneyMata

**DOI:** 10.1128/mra.00253-23

**Published:** 2023-05-31

**Authors:** Madison M. Moore, Mary A. Ayuk, Amber A. Johnson, Triniti Sims, Sanaa Haamen, Ramata Haidara, Adrian D. Allen, Leon A. Dickson, Somiranjan Ghosh, Ayele Gugssa, Hemayet Ullah, Glory B. Bassey, Lourds M. Fernando, Laricca Y. London, Esohe G. Irabor, Swagota D. Roy, Benedict K. Quagraine, Michael Smith, Howard University SEA-PHAGES Students, Winston A. Anderson, Courtney J. Robinson

**Affiliations:** a Department of Biology, Howard University, Washington, DC, USA; b Department of Biological and Environmental Sciences, Alabama A&M University, Normal, Alabama, USA; Queens College Department of Biology

## Abstract

Five subcluster C1 mycobacteriophages, Blackbrain, Cactojaque, Kboogie, Trinitium, and YoungMoneyMata, were isolated from soil using the host Mycobacterium smegmatis mc^2^155. The genome sizes range from 154,512 to 156,223 bp. The largest genome encodes 237 predicted proteins, 34 tRNAs, and 1 transfer-messenger RNA (tmRNA).

## ANNOUNCEMENT

With the goal of studying bacteriophage evolution and diversity, students in the Science Education Alliance—Phage Hunters Advancing Genomics and Evolutionary Sciences (SEA-PHAGES) program isolated five mycobacteriophages (Blackbrain, Cactojaque, Kboogie, Trinitium, and YoungMoneyMata) from soil collected from Howard University’s campus (Blackbrain and Cactojaque: 38°55′11.0″N, 77°01′10.0″W; Kboogie: 38°55′22.0″N, 77°01′07.0″W; Trinitium: 38°55′18.5″N, 77°01′09.0″W; and YoungMoneyMata: 38°55′21.7″N, 77°01′14.9″W) (1, 2).

As described previously, soil filtrates were generated by adding 7H9 broth to soil samples, shaking at 37°C for 2 h, and then filtering (pore size, 0.22 μm) (3). Mycobacterium smegmatis mc^2^155 cells were added to the filtrates, and the samples were incubated aerobically at 37°C for 48 h with shaking. The samples were then refiltered and plated with M. smegmatis mc^2^155 as the host. Three rounds of serial dilution were conducted for each plaque morphology of interest for purification. After single phage populations were obtained, genomic DNA was isolated from the cell lysate using the Promega Wizard DNA cleanup kit (2). Sequencing libraries were prepared using the Ultra II FS kit with dual-indexed barcoding (New England Biolabs). An Illumina MiSeq instrument was used to sequence the pooled libraries, yielding 150-base single-end reads (Blackbrain, 326,268; YoungMoneyMata, 178,427; Kboogie, 521,155; Trinitium, 167,263; and Cactojaque, 384,435). The genome reads were trimmed and assembled using Newbler v2.9, yielding a single contig for each genome. Identification of the genomic termini was completed using Consed v29.0 (4–6). The phages all had circularly permuted genomes.

DNA Master v5.23.3 (http://cobamide2.bio.pitt.edu/computer.htm) and the Phage Evidence Collection and Annotation Network (PECAAN) (7) were used to annotate the genomes (https://blog.kbrinsgd.org/overview/). The presence of genes and their starting points were predicted and selected using GLIMMER v3.0, GeneMark v2.5, and Starterator v1.1 (https://github.com/SEA-PHAGES/starterator) (8, 9). Predicted gene functions were assigned utilizing Phamerator Actino_Draft v402 (https://phamerator.org), BLAST v2.11.0+, PhagesDB, HHpred v3.0, and the NCBI Conserved Domain Database v3 (10–14). tRNAs and transfer-messenger RNAs (tmRNAs) were detected using ARAGON v1.2.38 and tRNAscan-SE v2.0 (15, 16). PhagesDB and the NCBI SRA Taxonomy Analysis tool were used to assign taxonomy and cluster affiliations based on nucleotide similarity (12, 17). Default parameters were used for all software. Genome information is available in Table 1.

**TABLE 1:** Genome characteristics and accession numbers of five C1 mycobacteriophages

Phage name	Sequencing coverage (×)	GenBank accession no.	SRA accession no.	Genome length (bp)	G+C content (%)	No. of genes (ORFs)^*a*^	No. of tRNAs	Top BLASTn match (GenBank accession no.; % identity)
Blackbrain	298	MK878897	SRX10013851	155,713	64.6	232	34	Cactojaque (MN428065.1; 99.83)
Cactojaque	354	MN428065	SRX10013856	154,918	64.6	228	34	Janiyra (MT818423.1; 99.67)^*b*^
Kboogie	473	MN234193	SRX10013841	155,680	64.7	237	34	Wally (JN699625.1; 99.74)
Trinitium	154	MN276192	SRX10013852	154,512	64.7	232	34	Quasimodo (NC_054721.1; 99.69)
YoungMoneyMata	265	MN183285	SRX10013855	156,223	64.7	235	33	JustHall (MK359333.1; 99.37)

a ORFs, open reading frames.

b Phage Janiyra’s genome comparison to Cactojaque’s genome resulted in a higher maximum score than the comparison to Blackbrain.

**FIG 1:**
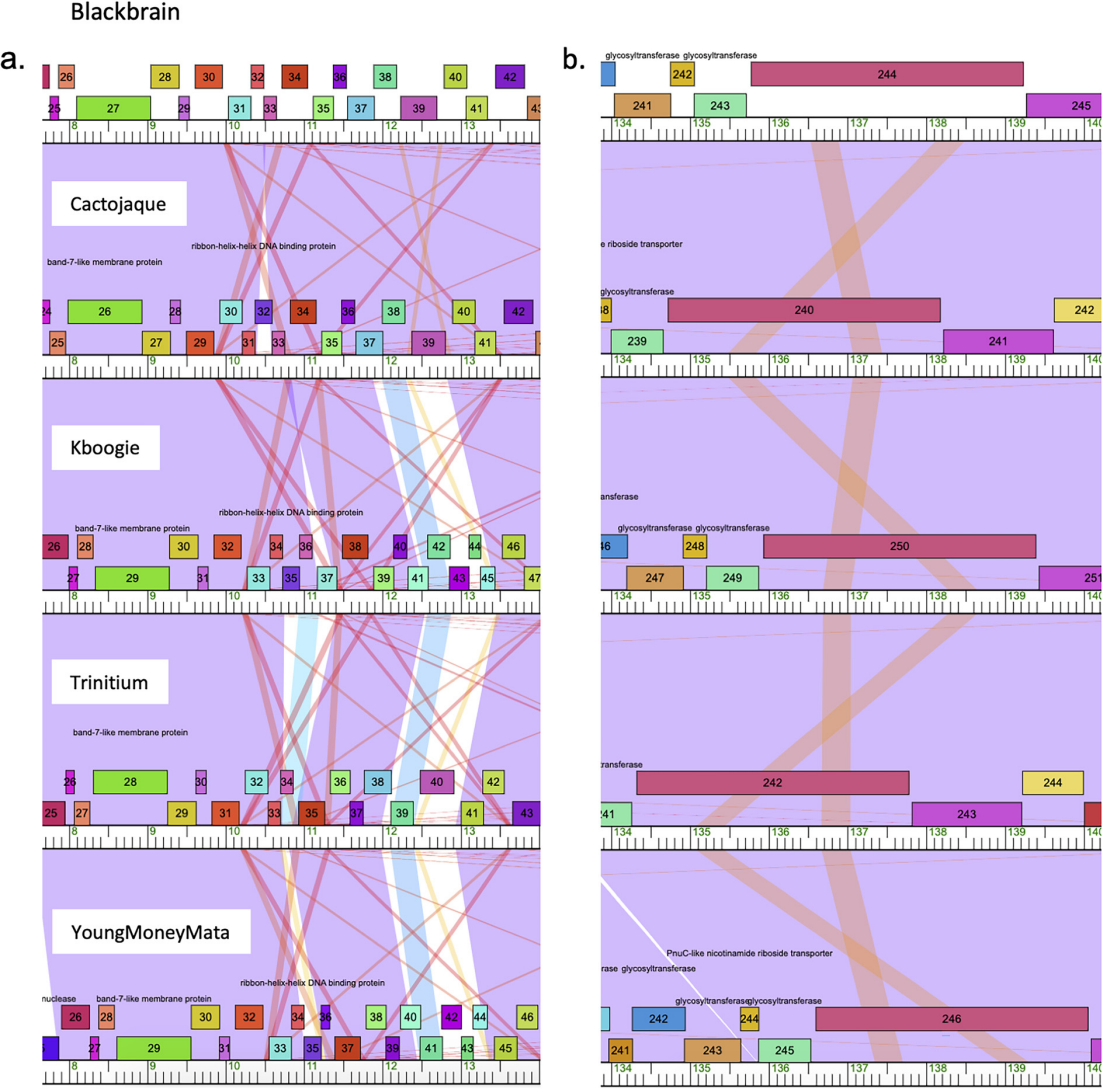
Excerpts of genome maps (7650bp to 14000bp (a) and 133,850bp to 141,000bp (b)) generated using Phamerator displaying areas of low nucleotide similarity in the genomes of phages Blackbrain, Cactojaque, Kboogie, Trinitium, and YoungMoneyMata (10). Violet shading on the maps indicates 100% nucleotide identity, while blue, yellow, and red indicate decreasing nucleotide identity and white signifies no nucleotide identity (10).

Nucleotide similarity analysis determined that the phages belong to subcluster C1 and have myovirus morphology (17). The genome sizes range from 154,512 to 156,223 bp (Table 1). The largest number of open reading frames was found in the Kboogie genome, which is the third largest of the genomes (Table 1). The genomic characteristics were consistent with other C1 genomes based on current PhagesDB entries, with each of the five having 1 tmRNA, 64.6% or 64.7% G+C content, and all except one having 34 tRNAs (Table 1) (12). Also consistent with other C1 phages, the genomes are highly similar to each other (18). Blackbrain and Cactojaque were most similar (nucleotide identity, 99.83%), and the most different genomes were YoungMoneyMata and Cactojaque (nucleotide identity, 99.19%). Despite the high nucleotide similarities across the genomes, Phamerator analysis revealed two distinct areas with multiple instances of low similarity (Fig. 1).

### Data availability.

The complete genome sequences and raw sequencing reads are available at GenBank and the NCBI Sequence Read Archives, respectively. The accession numbers are provided in Table 1.
